# Overfit detection method for deep neural networks trained to beamform ultrasound images

**DOI:** 10.1016/j.ultras.2024.107562

**Published:** 2024-12-27

**Authors:** Jiaxin Zhang, Muyinatu A. Lediju Bell

**Affiliations:** a Department of Electrical and Computer Engineering, Johns Hopkins University, Baltimore, MD, USA; b Department of Biomedical Engineering, Johns Hopkins University, Baltimore, MD, USA; c Department of Computer Science, Johns Hopkins University, Baltimore, MD, USA

**Keywords:** Deep learning, Image reconstruction, Ultrasound, Beamforming, Benchmarking, Standardization

## Abstract

Deep neural networks (DNNs) have remarkable potential to reconstruct ultrasound images. However, this promise can suffer from overfitting to training data, which is typically detected via loss function monitoring during an otherwise time-consuming training process or via access to new sources of test data. We present a method to detect overfitting with associated evaluation approaches that only require knowledge of a network architecture and associated trained weights. Three types of artificial DNN inputs (i.e., zeros, ones, and Gaussian noise), unseen during DNN training, were input to three DNNs designed for ultrasound image formation, trained on multi-site data, and submitted to the Challenge on Ultrasound Beamforming with Deep Learning (CUBDL). Overfitting was detected using these artificial DNN inputs. Qualitative and quantitative comparisons of DNN-created images to ground truth images immediately revealed signs of overfitting (e.g., zeros input produced mean output values ≥0.08, ones input produced mean output values ≤0.07, with corresponding image-to-image normalized correlations ≤0.8). The proposed approach is promising to detect overfitting without requiring lengthy network retraining or the curation of additional test data. Potential applications include sanity checks during federated learning, as well as optimization, security, public policy, regulation creation, and benchmarking.

## 1. Introduction

Medical images are often employed to non-invasively view the contents of the human body and render patient diagnoses. The formation of these medical images are typically governed by strict criteria to maintain accuracy and fidelity to the depicted anatomy [1]. The images may then be post-processed prior to display to remove noise or artifacts. Thus, each medical imaging method available in clinics today has a standard set of image formation or post-processing algorithms applied to create displayed images. Ultrasound imaging is one of the most common medical imaging modalities that abide by these criteria.

In ultrasound imaging, conventional image formation methods, such as delay-and-sum (DAS) beamforming, typically rely on known array geometries and medium properties [2]. The DAS beamforming method can be used to form real-time ultrasound images from raw radiofrequency (RF) channel data received after plane wave transmission (i.e., after targets of interest are insonified with one or more plane waves), with a trade-off between speed and image quality based on the number of transmitted plane waves. When compared to the traditional DAS algorithm, ultrasound image formation with deep learning is advantageous because networks can be trained to directly output high-quality images from raw sensor data, particularly after only a single plane-wave ultrasound transmission [3–10].

One common challenge when implementing deep neural networks (DNNs), both in the field of medical image formation and more broadly across many sectors of the deep learning arena, is the potential for overfitting. Overfitting is generally defined as the exact fit of the model to the training set, which is associated with the representation power of the model, regularization techniques, and optimization methods, and it is defined independently of the data size [11]. When overfitting occurs in ultrasound beamforming, networks may perform very well on training data, yet fail to generalize across different unseen datasets [12].

Common methods such as early stopping, k-fold cross-validation, or inference are widely adopted as effective approaches to prevent or detect overfitting [13,14]. In early stopping, training and validation errors are monitored, and validation errors are measured to represent generalization errors (i.e., the errors associated with predicting outcome values for previously unseen data). In addition, early stopping criteria are implemented to decide when to stop a training process and achieve minimum generalization loss. Traditional early stopping criteria include validation losses, quotient of validation losses, or progress exceeding a particular threshold [15]. Cross-validation is one of the most common methods to avoid network overfitting when there is a limited dataset [16]. In k-fold cross-validation [17], a dataset is split into *k* groups and the fitting and evaluation process (based on *k* − 1 training sets and 1 validation set) is performed *k* times. The final model skill score shows the generalization of the network quantitatively. Successful implementation relies on different data splits, which require multiple data samples [18]. With an inference approach to detect overfitting, additional test data are input to further evaluate DNN performance [19,20]. This additional ultrasound sensor data can be obtained through experiments or simulations, or from publicly available datasets.

Major limitations of the early stopping, cross-validation, and inference methods are that they require training data, re-training of the network, or curation of new test data. However, when presented with a new DNN without access to training code, training data, and unseen test data, implementation of these methods are not possible. In addition, considering that the training process typically requires thousands of training examples, it is not always feasible for a user to train a new DNN to perform the same task as that learned with an existing DNN to provide confidence that the network performs as expected. More recent advancements in overfitting detection techniques (e.g., adversarial examples [21], model selection [22], dynamic architectures [22]) suffer from these same challenges. These challenges are additionally concerning with respect to regulatory procedures [23], optimization, and trustworthiness of DNNs deployed on patient data.

In addition, when implementing federated learning [24–26] approaches to address privacy concerns [27,28], models are collaboratively trained across multiple local edge devices or servers holding decentralized local data samples. Federated learning can be realized with different workflows, including an aggregation server with centralized training topology [29] or peer-to-peer clients with decentralized training topology [30], without sharing training data between institutions. In each of these cases, it is most ideal if training code and data are not required to build confidence that an existing publicly available DNN will perform well on new data related to the trained task.

**Fig. 1.**
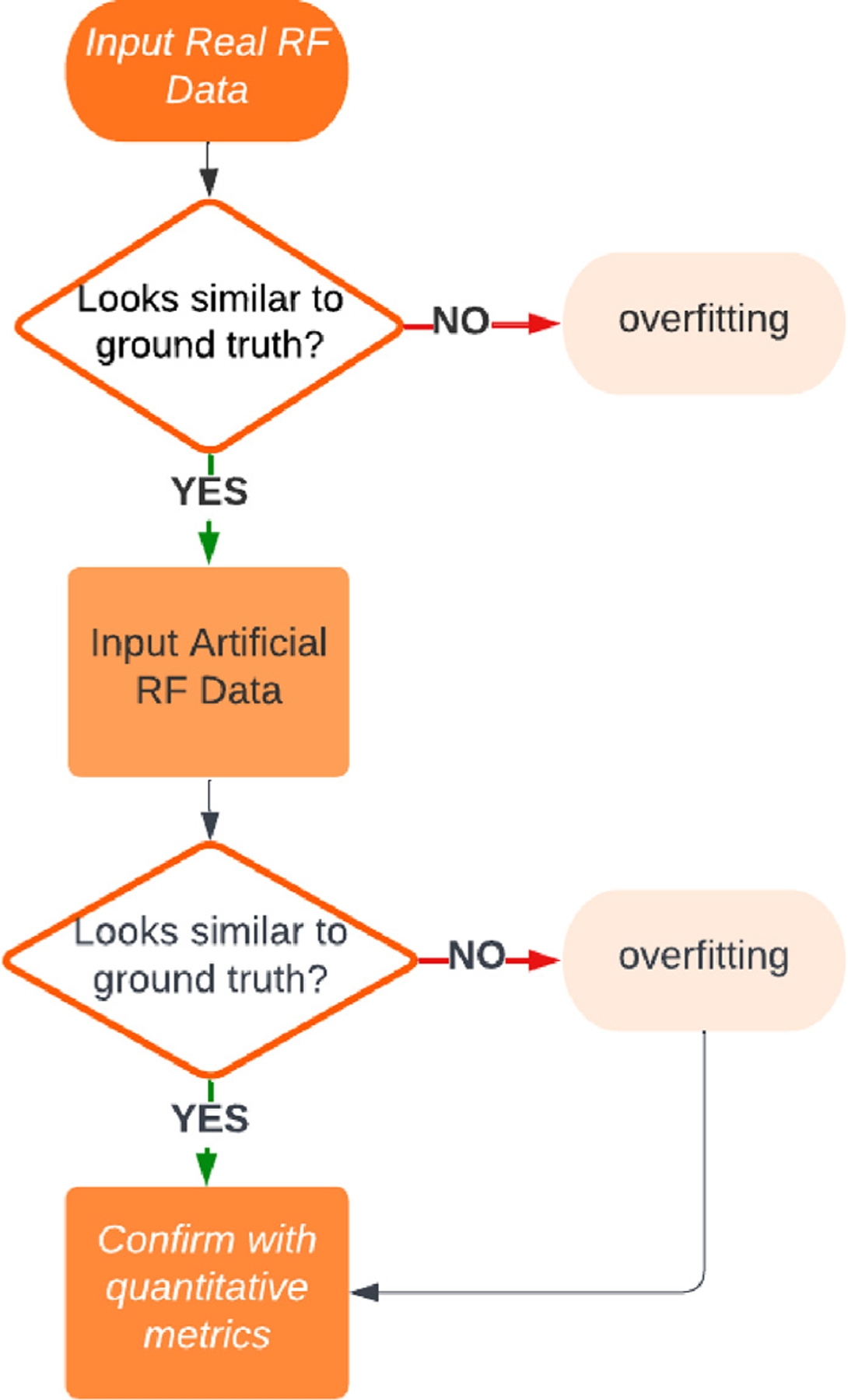
Flowchart describing when and how to deploy the proposed overfitting detection method.

In this paper, a novel method to more rapidly identify the overfitting of DNNs trained to beamform ultrasound images when compared to conventional overfitting detection approaches is proposed. The underlying premise is that a true beamformer should create images regardless of the input data being real or artificial, or previously unseen by the network. A DNN that recreates an image when presented with an artificial input should therefore produce the same type of image that would be produced by the ground truth beamformer after which the DNN is modeled. A preliminary report of this approach was presented in a conference paper [31]. Herein, new ultrasound image examples are included (e.g., *in vivo* examples to show the potential clinical impact), and all ultrasound image examples are now normalized to the same scale to achieve more accurate visual comparisons (which is a significant update to enable fair qualitative comparisons with respect to the qualitative assessment component of the proposed approach). In addition, we present a flowchart summarizing intended use (Fig. 1) and new results (including additional metrics and results obtained with progressive data removal from real to artificial RF data).

The proposed method does not require any training code, training data, or test examples. Thus, this method is effective when only provided with a DNN and its input data structure. As a result, users can employ the proposed method to determine if a DNN is overfitting before any testing on previously unseen data (which may not be readily available). Publicly available DNNs [5,6] and datasets [12,32,33] that anyone can use are implemented to validate the promise of the proposed method. In addition, the employed data and DNN models, training weights, and/or code originated from multiple institutions, which mimics the decentralization component of federated learning with no sharing of training data.

The remainder of this paper is organized as follows. Section 2 describes the artificial input data we propose, the DNNs investigated in this work, and the metrics for evaluating network performance. Section 3 presents images produced by the DNNs and corresponding qualitative and quantitative analyses. Section 4 discusses major findings and the associated implications for future implementation, and Section 5 summarizes major contributions.

## 2. Methods

### 2.1. Artificial RF data for ultrasound imaging

Robust networks generalize across different datasets while overfitted models perform well only on training data [34]. To test networks on unseen data, we created three types of artificial RF channel data based on the underlying premise stated in Section 1 to meet two basic criteria. First, the artificial data are expected to have never been seen by the networks because they do not resemble real data and should not be included during training. At the same time, the artificial data should be simple enough such that the associated image produced by a traditional beamformer is predictable and understandable. The artificial data we created meet these two criteria and are grouped into two categories: (1) binary samples including zeros and ones and (2) random samples.

With zeros as the input, the output envelope image contained zeros at each pixel location, resulting in invalid values after normalization. To obtain a valid output, a value close to zero (i.e., 1 × 10^−20^) was used instead. In addition, one RF channel data point at the center of the input was set to 1 to achieve a normalized image that was representative of the input and distinguishable from the second binary input. This second binary input was a matrix of ones surrounding a center pixel value of 1×10^−20^ to address the same normalization challenges described above.

A matrix of random samples drawn from a Gaussian distribution with mean *μ* = 0 and standard deviation *σ* = 1 was created to be the third type of artificial RF channel data. This type of data may resemble electronic noise obtained from an ultrasound transducer when no image target is present. To maintain the same range as the zeros and ones input data described above, the random input values were normalized to the range [0, 1].

### 2.2. Ground truth, test networks, and associated training/test data

The artificial channel data proposed in Section 2.1 were inputs to a Pytorch DAS plane-wave beamforming algorithm [12] and to three DNN models submitted by Rothlübbers et al. [5], Goudarzi et al. [6], and Wang et al. [7] to the Challenge on Ultrasound Beamforming with Deep Learning (CUBDL) [12,35]. For brevity, these three DNNs are referred to as Network A, Network B, and Network C, respectively (with architecture and associated training/test data summaries provided below, based on the details in [7,12]). The output single 0° plane wave images from the DAS beamformer served as the ground truth, as each of the three test networks were modeled after the DAS beamformer. Network C was flagged by the CUBDL organizers as overfitting to the training data during the evaluation phase for submitted networks, given its performance on previously unseen, crowd-sourced, test data [12], which is one of the currently subjective standards to determine overfitting, as described in Section 1.

Network A [5] is a fully convolutional network with four layers. The network was designed to model the united sign coherence factor (USCF) [36] by computing pixel-wise weighting (after data preprocessing, which included delay compensation and apodization [5]). The network input is time-delayed, magnitude-normalized, complex-valued data from the 0° plane wave transmission angle. The network output is a real-valued weighting factor for each reconstructed pixel. The final pixel values were obtained by multiplying the unweighted sum absolute pixel values by the network output pixel weights, followed by log compression and a correction for the maximum value. The integration of Network A into the beamforming pipeline and the network architecture are presented in Figs. 1 and 2 in [5]. This network used an Adam optimizer with a learning rate decay of 0.1 every 5 epochs, and it was trained for 15 epochs. The loss was computed as a linear combination of mean-squared error (MSE) and multiscale structural similarity (MS-SSIM) [37] loss on the log compressed, normalized final images.

Training data for Network A consisted of 107 ultrasound raw data sets of a phantom (Model 054GS, CIRS, Norfolk, VA), acquired with multiple angles using a 128-element linear array transducer (DiPhAS, Fraunhofer IBMT, Sankt Ingbert, Germany). High-quality target images were reconstructed using multi-angle USCF imaging [36], utilizing data from seven plane wave angles. The publicly available Plane-wave Imaging Challenge for Medical Ultrasound (PICMUS) dataset [33] was used to test the model.

Network B [6] utilizes the MobileNetV2 [38] architecture, as presented in Fig. 2 in [6]. The network was designed to estimate and apply an apodization window to the input in-phase and quadrature (IQ) channel data for minimum variance beamforming [39] (after data preprocessing, which included delay and f-number compensation [6]). The network input is a 2 × *m* × *n* matrix in which first the two channels are the real and imaginary parts of IQ data, *n* is the number of channels, and *m* is the length of the window considered for temporal averaging to preserve the speckle statistics. The network output is a two-dimensional vector containing real and imaginary parts of the beamformed data. The output IQ data was then envelope detected and log compressed to obtain the final B-mode ultrasound image. This network used an AdamW optimizer [40]. The loss was computed as the $\ell_{1}$-norm betweenthe network output and the IQ pixel values obtained using minimum variance beamforming.

Training data for Network B consisted of the publicly available plane wave and focused transmission phantom,*in vivo*, and Field II-simulated datasets available in the Ultrasound Toolbox [33,41,42]. The plane wave data were acquired with a Verasonics (Kirkland, WA, USA) Vantage 256 scanner and L11–4v probe (phantom and *in vivo* data) or an Alpinion (Seoul, South Korea) E-Cube12R scanner and L3–8 probe (phantom data). Focused imaging datasets were acquired with a Verasonics Vantage 256 scanner connected to a P4–2v probe and an Alpinion E-Cube12R scanner connected to a L3–8 probe. Images reconstructed from data received after a single 0° plane wave transmission were the ground truth output images utilized during training.

Network C [7] is a conditional generative adversarial network (cGAN) [43] consisting of one generator and two discriminators, designed to directly transform RF channel data to a B-mode ultrasound image. As presented in Fig. 1 in [7], the generator architecture is based on U-Net [44], and the discriminator has an analogous design to the contraction path, which was implemented twice to calculate the cross-entropy loss between the input, ground truth, and generated images. Adam optimization was applied with 800 epochs for pre-training, 800 epochs for training, and 200 epochs for fine-tuning, while the initial learning rate was 0.0002.

Pre-training data for Network C consisted of 400 photographs from the CMP Facades datasets [45], and the training data consisted of 1500 single plane wave ultrasound images from PICMUS [33] and the Ultrasound Toolbox [41]. In addition, −2 dB Gaussian white noise was added to each single plane wave RF signal. The ground truth images for training were formed after incorporating the 75 plane wave transmissions to create each corresponding DAS image. The entire dataset was divided into dedicated training (60%), validation (20%), and test (20%) datasets.

While there are multiple differences in the design and training processes for Networks A, B, and C, two major differences emerge based on the published reports (notwithstanding the architecture differences summarized above). First, Networks A and B learned weights (e.g., scaling, apodization) to be included in an otherwise traditional DAS beamforming process, whereas Network C was designed to directly transform RF channel data to a B-mode ultrasound image. Second, Network C was pre-trained with examples from real-world photographs (consisting of building facades), whereas Networks A and B appear to have been exclusively trained using ultrasound data.

### 2.3. Evaluation methods to detect overfitting

Qualitative assessment and two classes of quantitative metrics were employed to identify overfitting with the artificial inputs described in Section 2.1, as summarized by the decision tree in Fig. 1. Initially, qualitative assessment should be performed to determine similarity to the ground truth. Results that are similar pass the first checkpoint and are not suspected to be overfitting to the training data.

When the artificial inputs are introduced for additional evaluation and assessment, the first class of quantitative metrics for each DNN output and corresponding ground truth is the mean ± one standard deviation of the envelope-detected ultrasound images. With the zeros input, we expect the mean produced by this evaluation metric to be ≈0 (i.e., close to the ground truth zero mean result), unless the network is overfitting. With the ones input, the mean pixel values of the ground truth and DNN outputs are expected to be ≈1, unless the network is overfitting. Similarly, with the Gaussian random input, we expect the mean values to be close to that of the ground truth.

The second class of quantitative metrics for images that pass the two checkpoints described above is an image-to-image comparison based on $\ell_{1}$ and $\ell_{2}$ losses:

(1) $$\ell_{1}=\frac{1}{N}\sum_{n=1}^{N}\left|x_{n}-y_{n}\right|$$

(2) $$\ell_{2}=\sqrt{\frac{1}{N}\sum_{n=1}^{N}{\left|x_{n}-y_{n}\right|}^{2}}$$

where x and y denote the normalized DAS reconstructed ground truth and the DNN output images, respectively, and N is the total number of overlapping pixels evaluated when comparing the two images. Two additional image-to-image metrics that consider specific patterns or structures include the normalized cross correlation (NCC) [12]:

(3) $$\text{NCC=}\frac{\sum_{n}\left(x_{n}-\mu_{x}\right)\left(y_{n}-\mu_{y}\right)}{\sqrt{\left({\sum_{n}\left|x_{n}-\mu_{x}\right|}^{2}\right)\left({\sum_{n}\left|y_{n}-\mu_{y}\right|}^{2}\right)}}$$

where μ represents the mean of the image data, and the structural similarity index measure (SSIM) [46,47]:

(4) $$\begin{array}{c} \text{SSIM}\;=\;l(x,y)c(x,y)s(x,y)\\ \;=(\frac{2\mu_{x}\mu_{y}+C_{1}}{\mu_{x}^{2}+\mu_{y}^{2}+C_{1}})(\frac{2\sigma_{x}\sigma_{y}+C_{2}}{\sigma_{x}^{2}+\sigma_{y}^{2}+C_{2}})(\frac{\sigma_{xy}+C_{3}}{\sigma_{x}\sigma_{y}+C_{3}})\\ \;=\frac{\left(2\mu_{x}\mu_{y}+C_{1})(2\sigma_{xy}+C_{2}\right)}{\left(\mu_{x}^{2}+\mu_{y}^{2}+C_{1})(\sigma_{x}^{2}+\sigma_{y}^{2}+C_{2}\right)} \end{array}$$

where σ represents the standard deviation of the image data; $l\left(x,y\right)$, $c\left(x,y\right)$, and $s\left(x,y\right)$ are luminance, contrast, and structure comparison functions, respectively; and $C_{1}$, $C_{2}$, and $C_{3}=C_{2}/2$ are positive constants used to avoid null denominators and are computed based on the dynamic range of the image. We additionally report NCC and SSIM between the outputs of Network C obtained with the proposed artificial inputs and the output of the Pytorch DAS beamformer [12] when inputting channel data from a PICMUS image of a CIRS Model 040GSE Phantom obtained with 75 plane wave transmissions (which Network C learned well and appears to overfit).

### 2.4. Validation with progressive data removal

To validate network performance expectations as inputs transition from realistic image targets to unrealistic patterns, PICMUS [33] channel data acquired from a CIRS Model 040GSE Phantom were progressively removed from real channel data received using the entire 128 transducer elements (i.e., 0% removal), in 5% increments. At each increment percentage, a subset of transducer element locations were randomly selected for removal, after rounding to the nearest integer. The removed channel data associated with the selected elements were replaced with near-zero values (i.e., 1 × 10^−20^). At 100% removal, the artificial zeros input described in Section 2.1 was employed. DNN-generated images were compared to the corresponding ground truth at each step, both qualitatively and quantitatively using the metrics that consider specific patterns (i.e., NCC, SSIM). The quantitative metrics were plotted as functions of the progressively removed data percentage.

### 2.5. Calculating the number of trainable parameters

To determine the number of trainable parameters used to evaluate network complexity and provide insight into the potential for overfitting, the same method implemented by Hyun et al. [12] was employed. In particular, the number of learnable parameters in each layer corresponds to the number of weights and biases in each network, which are determined by the number of neurons for a fully connected layer and the number and the size of filters for a convolutional layer [48, 49]. These parameters require gradient computations, resulting in the greater model complexity of neural networks than the conventional DAS beamforming approach.

## 3. Results

### 3.1. Initial evaluation

**Fig. 2.**
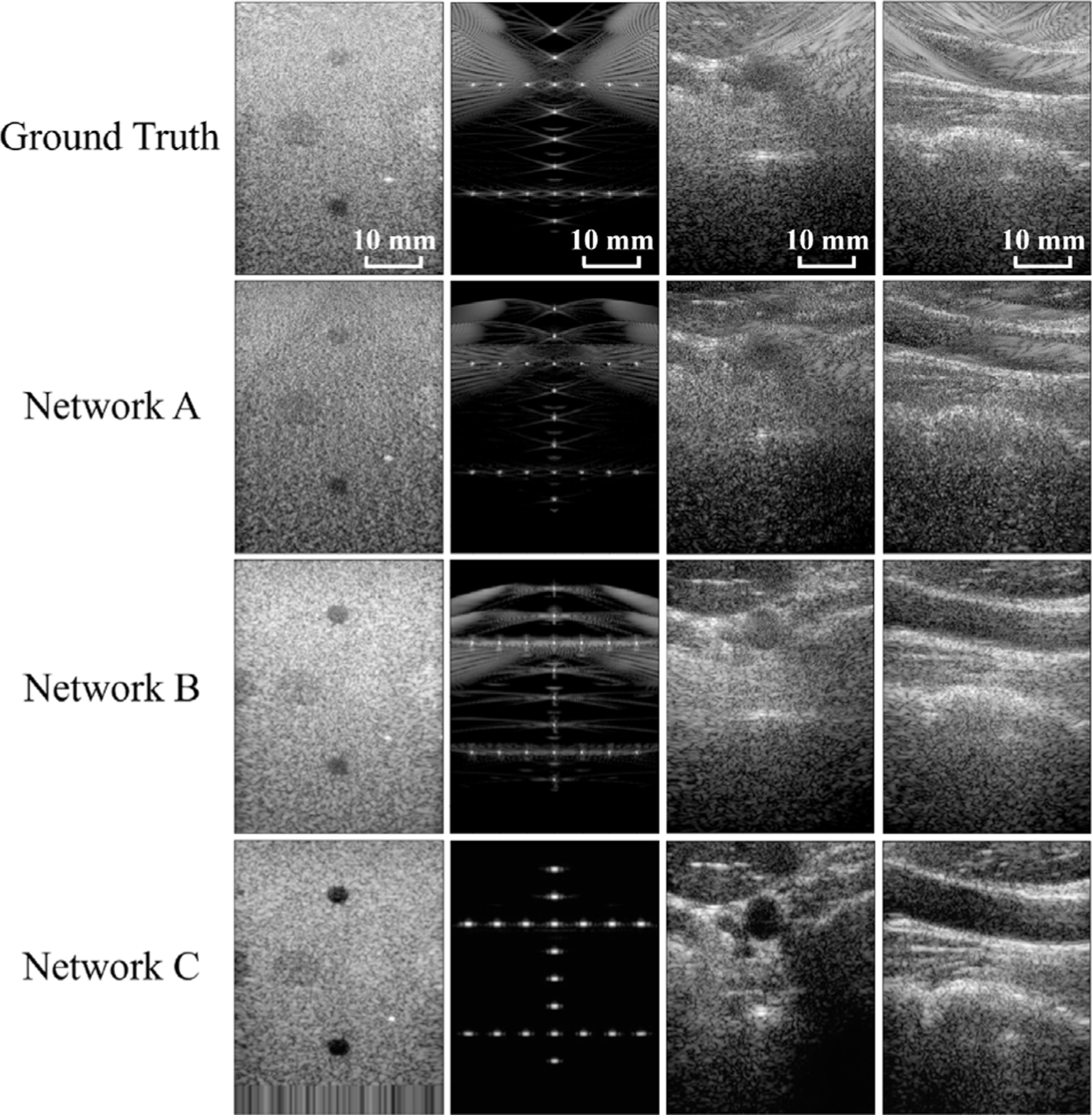
Baseline evaluation on PICMUS data [33] acquired from a CIRS Model 040GSE Phantom (first column), Field II [50,51] simulated data (second column), and orthogonal cross sections of an *in vivo* carotid artery (third and fourth column). Images are displayed with 60 dB dynamic range.

Fig. 2 shows log-compressed ultrasound B-mode images created with the publicly available PICMUS data [33]. These PICMUS data were employed to train Network C (in tandem with simulated ultrasound data) [7], while Network A was trained on data acquired by Rothlübbers et al. [5], and Network B was trained on public data available with the Ultrasound Toolbox [6,41]. The network-produced images were similar to their respective ground truths, confirming that the networks and data were correctly loaded. In particular, Network C performed well on the dataset used for training of this DNN and generated cleaner images than the ground truth. Without the additional analysis outlined in Fig. 1, it remains a question as to whether this is a true improvement or simply a reflection of overfitting.

### 3.2. Zeros input

**Fig. 3.**
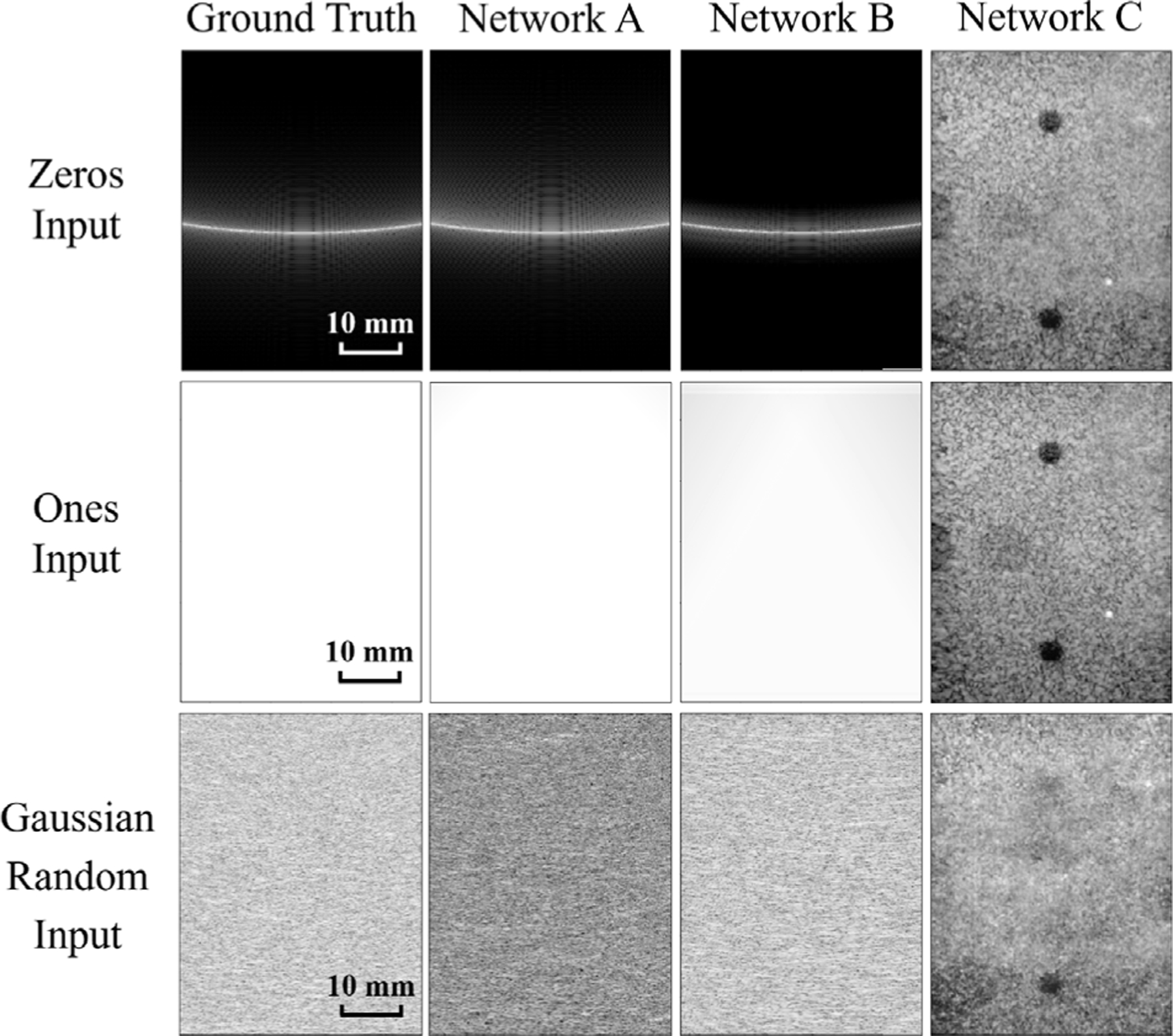
Network-produced images with artificial radiofrequency channel data inputs, including zeros (top), ones (middle), and Gaussian noise (bottom). Images are displayed with 60 dB dynamic range.

The top row of Fig. 3 shows the proposed method employed to reveal the answer to the quandary regarding improvement vs. overfitting, starting with an input of mostly zeros surrounded by a single pixel containing a value of 1 (i.e., zeros input). Networks A and B produced images that look similar to the ground truth. In particular, the point spread function (PSF) of the singular center pixel with a value of 1 seems to be represented. However, Network C did not replicate the ground truth PSF and instead created an image that is similar to its training data (see top left of Fig. 2).

Table 1 reports the mean ± one standard deviation of the envelope-detected ultrasound image output. The mean and standard deviation of pixel values in images created with the zeros input are generally similar to their respective ground truths, with the exception of Network C, which produces values that have the greatest deviation from the ground truth (e.g., mean values ≥0.08, rather than values closer to a mean of zero). Table 2 reports $\ell_{1}$ and $\ell_{2}$ losses between ground truth and network-produced images. While these losses are minimal with Networks A and B, Network C produced an image that has the largest $\ell_{1}$ and $\ell_{2}$ losses among the three networks. With the proposed zeros input method, the qualitative results in Fig. 3 and the quantitative results in Tables 1 and 2 demonstrate that Network C is overfitting to the training data.

### 3.3. Ones input

The middle row of Fig. 3 shows output images generated with the ones input. Networks A and B generated images that look like the ground truth, containing a similar all-white appearance when each image is displayed with the same dynamic range. However, Network C created an image similar to one of its training data (see Fig. 2) without reproducing the ground truth.

With the ones input, the mean values of envelope-detected images generated by Networks A and B are similar to that of the ground truth, which is close to one, as shown in Table 1, although the standard deviations show greater deviations when compared to that of the ground truth. The output image of Network C has a mean value that shows the greatest deviation from the ground truth (e.g., mean values ≤0.07, rather than values closer to a mean of one). Table 2 shows that Network C produced an image that has the largest $\ell_{1}$ and $\ell_{2}$ losses among the three neural networks, indicating the worst match between the output image of Network C and the ground truth. These qualitative observations and the associated quantitative analyses (i.e., mean, $\ell_{1}$, and $\ell_{2}$) reveal overfitting of Network C when assessed with the ones input.

### 3.4. Gaussian random input

The bottom row of Fig. 3 shows the output B-mode images with the Gaussian random input. Networks A and B produced images with similar appearance to the ground truth while Network C created an image that looks like its associated training data (see Fig. 2).

With the Gaussian random input, the mean ± standard deviation of the envelope-detected image produced by Network B is similar to that of the ground truth while Networks A and C both generated images with greater deviations from the ground truth, as shown in Table 1. The last column of Table 2 reports the largest $\ell_{1}$ and $\ell_{2}$ losses between ground truth and Network C among the three networks. These results obtained with the Gaussian random input show that the mean ± standard deviation measurement is not a suitable metric to identify overfitting with the Gaussian random input, and qualitative observations and $\ell_{1}$ and $\ell_{2}$ comparisons are more useful in this case. In addition, specific values to expect with the mean, $\ell_{1}$, or $\ell_{2}$ comparisons can be inconclusive for this type of input.

### 3.5. Evaluation of structural patterns

While the proposed approach is most concerned with DNN outputs that provide seemingly believable ultrasound images (until alternative artificial or real data inputs prove otherwise), the mean, $\ell_{1}$, or $\ell_{2}$ metrics do not completely represent structural or pattern differences in the data (e.g., leading to qualitative evaluations being more representative of overfitting with the Gaussian random input, as discussed in Section 3.4). The results from two additional metrics to address this concern are reported in Table 3, when comparing the expected ground truth output to the output achieved with each artificial input. Among the three tested networks, these NCC and SSIM metrics show that Network C consistently produces images with the worst match to the ground truth (i.e., ≤0.8 and ≤0.2, respectively).

The fourth and eighth rows of Table 3 additionally report the NCC and SSIM between the image outputs of Network C obtained with artificial inputs and the associated 75-plane-wave PICMUS image created with a DAS beamformer which Network C appears to overfit (as observed from Figs. 2 and 3). These NCC and SSIM results are greater than corresponding values achieved when comparing the ground truth outputs with the Network C outputs, which provides quantitative confirmation that Network C produces images that more closely resemble the structural patterns in this particular training example, rather than the otherwise expected ground truth output if overfitting were not present.

### 3.6. Progressive data removal

**Fig. 4.**
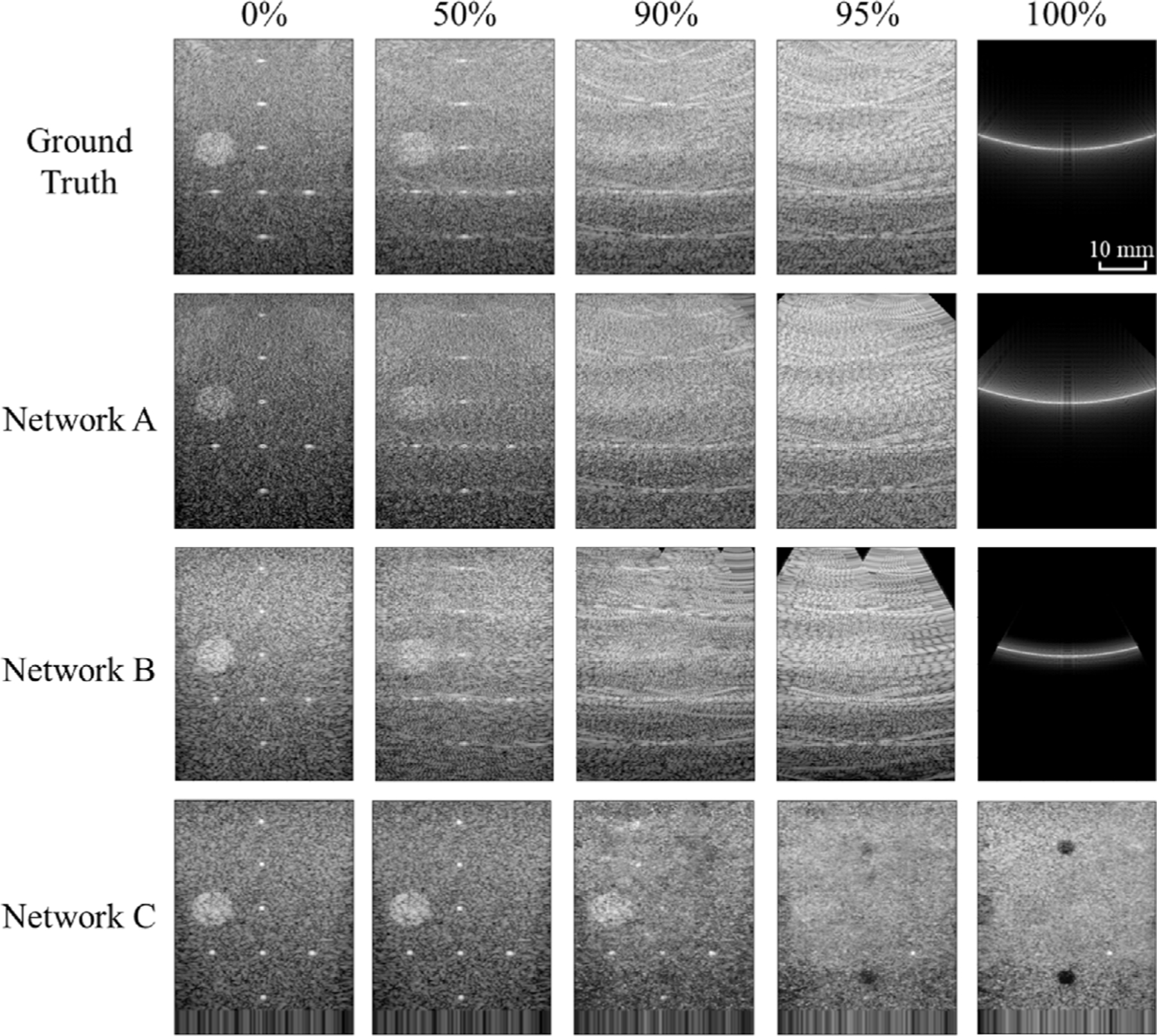
Ground truth and network-produced images with 0%, 50%, 90%, and 95%, of channel data removed, followed by the zeros input (considered to 100% of channel data removed). Images are displayed with 60 dB dynamic range.

Fig. 4 shows example ground truth and network-generated images when gradually removing channel data at random transducer element locations. From left to right, images progressively degrade with Networks A and B, as the percentage of data removed increases, which is expected. However, Network C produces images that seemingly maintain robustness relative to the initial (i.e., 0% removed) ground truth data, as the percentage of data removed increases. Rather than maintaining or breaking this seemingly excellent performance when presented with less data (e.g., at 90% data removal), Network C instead produces another previously learned pattern (i.e., one that is similar to another PICMUS phantom image included in the training data of Network C). In addition, with Network A or B, the structure of the input data (i.e., retaining entire RF channel data lines when removing data from 0% to 95% vs. retaining a single high-amplitude pixel at the center of the zeros input when transitioning from 95% to 100% removal) is responsible for the appearance of the output images. One similar feature across Networks A and B is the PSF caused by singular high-amplitude information (e.g., associated with point targets in the 0% images), while data pre-processing (e.g., apodization, f-number compensation) and element directivity patterns are likely responsible for the underlying angular patterns output by Networks A and B. Network C does not produce the same underlying patterns because it more directly transforms RF channel data into a B-mode ultrasound image.

**Fig. 5.**
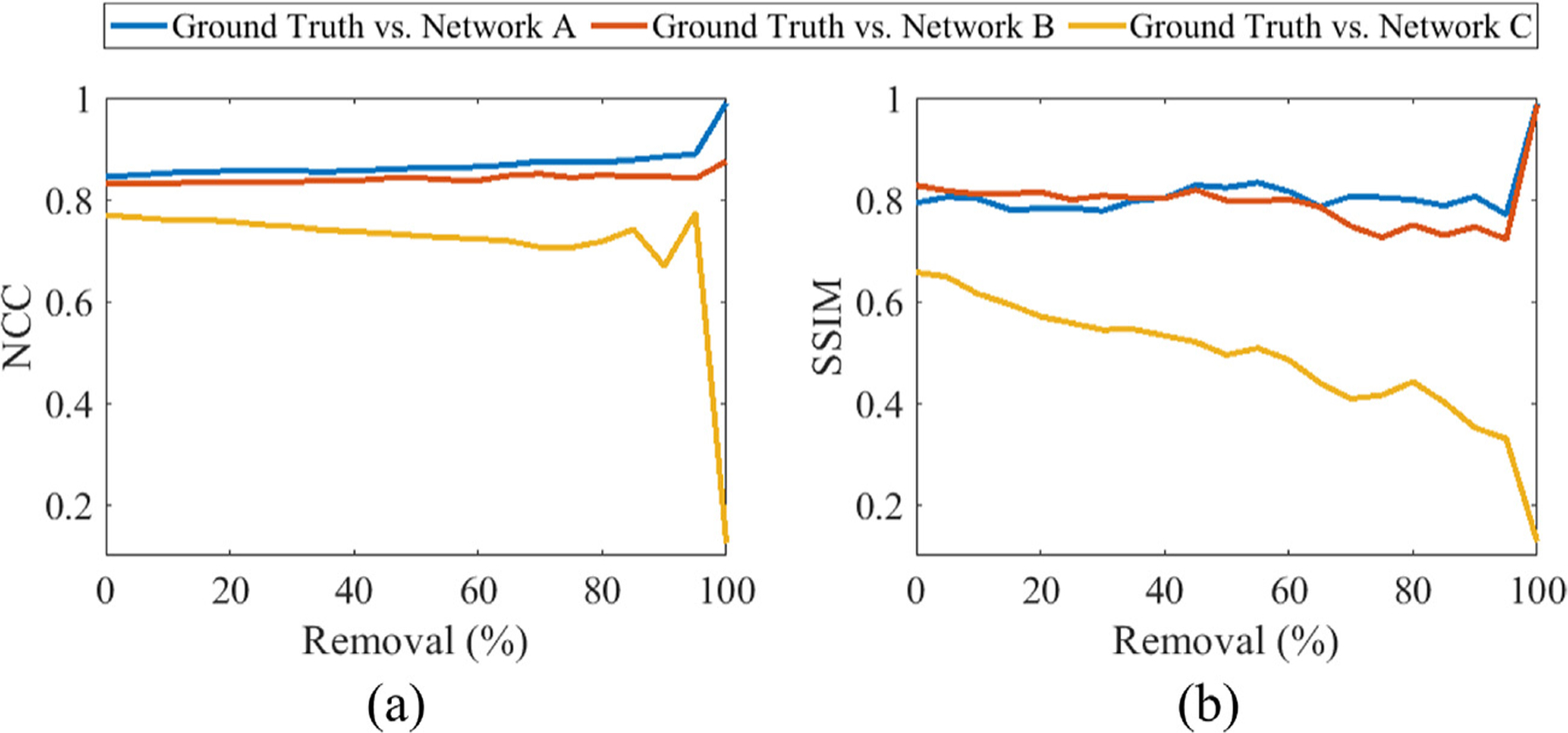
(a) Normalized cross correlation (NCC) and (b) structural similarity index measure (SSIM) as functions of the percentage of data progressively removed.

Fig. 5 shows NCC and SSIM as functions of channel data removal percentages. As the input data are progressively removed from 0% to 95%, the NCC and SSIM between ground truth and network-produced images are relatively constant with Networks A and B, then increase when the networks are presented with the zeros input at 100% removal. However, with Network C, these values decrease with an increase in removed data, followed by an additional decrease when this network is presented with the zeros input at 100% removal. It is a stark contrast that at 100% removal, Networks A and B generate images with the greatest NCC and SSIM among the removal process whereas Network C creates an image with the lowest NCC and SSIM, indicating the greatest deviation from the ground truth.

### 3.7. Number of learned parameters

Network complexity was assessed based on the number of learned parameters, including numbers of weights and biases in each layer for each network. In particular, we compared the number of trainable parameters of each neural network with that of the ground truth method. The method described in Section 2.3 was applied to obtain the number of learnable parameters for Networks A through C, with corresponding values reported in Table 4. Network C has 1–4 orders of magnitude more trainable parameters compared to those of Networks A and B.

## 4. Discussion

With the goal of rapidly identifying DNN overfitting, this work is the first to introduce a new type of analysis using artificial data as the input to DNNs trained to output ultrasound images. Three types of artificial ultrasound sensor data revealed overfitting of an ultrasound image reconstruction network (i.e., ones, zeros, and random). Overfitting was rapidly identified by qualitative observations. Quantitatively, overfitting was rapidly identified based on the largest $\ell_{1}$ and $\ell_{2}$ losses and the smallest NCC and SSIM between the network-produced images and the ground truth results, after inputting the artificial sensor data comprised of binary or Gaussian random values. This rapid overfitting identification was confirmed with the NCC and SSIM between the suspected overfit training example and the Network C outputs with artificial inputs (Table 3), followed by a systematic demonstration of persistent overfit results achieved with imposed data loss (Figs. 4 and 5).

The NCC metric generally provides the most conclusive and significant interpretation in terms of values to expect when implementing the approach proposed in Fig. 1, as values closer to 1 consistently indicate greater similarity with the underlying patterns. This effectiveness benefits from the invariance of NCC to linear brightness and contrast variations [52]. With the binary image input (i.e., zeros and ones), overfitting was additionally identified based on the greatest difference in mean pixel values between the network output and the ground truth (Table 1). However, this metric failed to inform overfitting with the Gaussian noise input, likely due to the absence of structural considerations with the mean values and also no clear expected difference between the means of the ground truth output and the DNN output. As noted above, overfitting was successfully identified with the Gaussian noise output, followed by understandable interpretation of the associated NCC results (Table 3 and Fig. 5), unlike interpretation of the mean result (Table 1). Hence, among the metrics presented herein, NCC is considered to be most suitable for this task. More generally, metrics that consider structural patterns (including NCC and SSIM) are well-suited for quantitative confirmation of overfitting assessments with artificial inputs that produce subtle output patterns.

The proposed overfitting approach is promising because it does not require a time-consuming retraining process using the training code and training data or the collection of additional test data. Instead, images produced by existing DNNs were evaluated after inputting the proposed artificial sensor data (i.e., ultrasound channel data) to provide more rapid identification of network overfitting when compared to traditional overfitting detection approaches. While simpler than simulating or curating large ultrasound datasets for testing (which was the approach implemented by the CUBDL organizers to arrive at the same overfitting conclusion for Network C [12,53]), the proposed approach (i.e., inputting artificial channel data consisting of zeros, ones, or Gaussian noise) is otherwise conceptually similar to inputting real ultrasound channel data. Progressive data removal (Figs. 4 and 5) additionally supports the overfitting conclusions determined by the CUBDL organizers, particularly when the evaluations herein were performed with the more interpretable and conclusive NCC metric.

As reported in Table 4, the network that was identified as overfitting with the approach presented in Fig. 1(i.e., Network C) has 1–4 orders of magnitude more trainable parameters compared to those of the other networks (i.e., Networks A and B). While there are various reasons why DNNs may overfit to training data, networks with greater complexity tend to have greater overfitting potential [54]. Therefore, this greater complexity is one possible reason for the observed overfitting. Another potential reason for the overfitting susceptibility is that Network C learned the entire beamforming process (i.e., from raw data to image output), while Networks A and B learned weights applied to a subset of this entire beamforming process.

Recognizing the statistical distinction between overfitting and generalization, while an overfit network may correlate with poor generalization performance, poor generalization performance does not necessarily correlate with overfitting [55]. However, in the context of beamforming, a network that is purported to beamform raw data should be capable of generalizing while avoiding overfitting. Thus, the two terms can be considered interchangeable in this context. From this perspective, additional causes of overfitting include limitations in the representation power of a model, the amount of training data utilized, and insufficient computational resources to avoid optimization errors [11].

One limitation of the proposed approach is that the artificial input datasets used in this manuscript (i.e., zeros, ones, and Gaussian noise) could potentially be incorporated in the training process, unbeknownst to the user or evaluator who did not develop the associated network. In this case, we encourage the development of unique artificial patterns by the individual performing the proposed approach. From this perspective, the proposed approach additionally has the potential to address concerns regarding transparency when local training data are kept private for federated learning [25,30], yet accurate testing is necessary. In particular, the proposed artificial input approach (e.g., zeros, ones, Gaussian noise, or any desired pattern combination that may be introduced in the future) relies on ensuring that these type of unrealistic inputs are never included in the private training data.

It may also be considered a limitation that our method lacks the ability to determine the level of overfitting when the output image is a combination of patterns from training data (e.g., Fig. 4, Network C), rather than an exact replica of one of the training images (e.g., Fig. 3, Network C). There can potentially be similar performance concerns associated with Networks A and B, based on three observations of the quantitative results reported in Tables 1–3. In Table 1, the mean of the output image of Network A obtained with the Gaussian noise input deviates more from the ground truth than that of Network B, whereas the mean of output image of Network B obtained with the ones input deviates more from the ground truth than that of Network A. In Table 2, with the ones input, Network A produced an image with smaller $\ell_{1}$ and $\ell_{2}$ losses relative to Network B, whereas with the Gaussian noise input, Network B created an image with smaller $\ell_{1}$ and $\ell_{2}$ losses than Network A. In Table 3, Network A produced images with higher NCC with the zeros input and higher SSIM with the ones input, relative to Network B. However, with the Gaussian noise input, Network B created an image with higher SSIM relative to that of Network A. Based on these observations, there are potentially minor performance concerns with Networks A and B, which may be more nuanced compared to the more obvious overfitting observations achieved with Network C when implementing our proposed method. Therefore, future work that quantifies the level of overfitting can potentially provide a more comprehensive analysis toward this end, after implementing new variations of the proposed approach.

Additional future applications and extensions of this approach require the consideration of known principles regarding image formation physics for ultrasound or other types of medical images. With a similar linear array sensor to that employed in ultrasound imaging, it is notable that a DNN designed to learn coherence-based beamforming [56–58], then adapted to photoacoustic imaging [59], successfully survived our proposed approach to demonstrate the absence of overfitting [59]. This achievement highlights the promise of extending the proposed approach to multiple beamforming applications. When implementing the proposed approach for different imaging modalities with sensors and physics that differ from ultrasound (and photoacoustic) imaging and sensing principles, the applicable artificial input data can potentially vary. In addition, the proposed approach has the potential to provide a new layer of oversight and benchmarking for regulatory bodies tasked with approving the deployment of DNNs on patient data.

### 5. Conclusion

This paper demonstrates applications of a novel method to rapidly identify overfitting of DNNs trained to beamform ultrasound images. The proposed approach consists of inputting artificial raw sensor data into DNNs and comparing the outputs with ground truth images. This approach does not require a time-consuming retraining process using the training code and training data nor the collection of additional test data. The artificial inputs must never be included in the training process to ensure success of the proposed approach. Results demonstrate that the proposed method is promising to be used as a general evaluation approach to identify DNNs that may have unexpectedly overfit to example input data that the networks were trained to reconstruct. Potential applications include sanity checks during federated learning, as well as optimization, security, public policy, regulation creation, and benchmarking.

## Figures and Tables

**Table 1:** Mean±one standard deviation of envelope-detected ultrasound data.

	Zeros	Ones	Gaussian noise
Ground truth	0.0052 ± 0.0363	0.9998 ± 0.0013	0.2326 ± 0.1238
Network A	0.0068 ± 0.0383	0.9953 ± 0.0237	0.0858 ± 0.0676
Network B	0.0034 ± 0.0334	0.8186 ± 0.1063	0.2366 ± 0.1298
Network C	0.0871 ± 0.0627	0.0619 ± 0.0460	0.0966 ± 0.0702

**Table 2:** $\ell_{1}$ and $\ell_{2}$ losses between ground truth and network-produced ultrasound data.

		Zeros	Ones	Gaussian noise
$\ell_{1}$ loss	Ground truth vs. Network A	0.0018	0.0045	0.1653
	Ground truth vs. Network B	0.0018	0.1812	0.1403
	Ground truth vs. Network C	0.0718	0.9394	0.1849

$\ell_{2}$ loss	Ground truth vs. Network A	3.20 × 10^−5^	0.0005	0.0415
	Ground truth vs. Network B	3.16 × 10^−5^	0.0040	0.0316
	Ground truth vs. Network C	0.0087	0.8846	0.0492

**Table 3:** NCC and SSIM between ground truth and network-produced ultrasound data.

		Zeros	Ones	Gaussian noise
NCC	Ground truth vs. Network A	0.9903	0.9480	0.8725
	Ground truth vs. Network B	0.8795	0.9398	0.8695
	Ground truth vs. Network C	0.1325	0.7893	0.7013
	PICMUS Phantom vs. Network C	0.8861	0.8250	0.7951

SSIM	Ground truth vs. Network A	0.9776	0.8651	0.2852
	Ground truth vs. Network B	0.9715	0.7933	0.4198
	Ground truth vs. Network C	0.1030	0.0658	0.0313
	PICMUS Phantom vs. Network C	0.6702	0.5434	0.4101

**Table 4:** Total number of trainable parameters in test networks.

	# of Parameters
Ground truth	0
Network A	3059
Network B	2,226,146
Network C	54,408,833

## Data Availability

All data and code are publicly available.
